# From authenticity ecosystems to human–AI hybrid societies

**DOI:** 10.1073/pnas.2631482123

**Published:** 2026-10-22

**Authors:** Ofer Tchernichovski, Nori Jacoby

**Affiliations:** ^a^ Department of Psychology, Hunter College and the Graduate Center, City University of New York, New York, NY 10021; ^b^ Department of Psychology, Cornell University, Ithaca, NY 14853

Fears of AI catastrophe imagine armies of misaligned agents coordinating to destroy humanity. In this issue, Zhang et al. (1) document something more mundane but immediately consequential: AI agents posing as humans are already corrupting information systems through which modern societies observe themselves. The people deploying them are survey participants trying to earn a few dollars by having an agent do their work. The agents have no incentive to cause harm and no intention to deceive anyone about the price of shampoo. They degrade our information lifelines simply because they are not here. An AI agent can answer a survey about shampoo coherently, fluently, and at length, but it has never used it.

Detachment from reality in online opinion surveys is invisible in the output, as it propagates silently into real-world decisions, including critical governance functions. A great deal of evidence-based decision-making rests on those surveys. They guide policymakers, public health officials, NGOs, and city planners by detecting shifts in social measures such as labor vulnerability, infrastructure demand, vaccine hesitancy, or housing stress. They also guide producers to tailor their products to their local consumers. Detecting AI bots early on in a survey can help survey makers in removing responders that are not human. However, previous studies have shown that AI agents can evade standard survey defenses, including attention checks, tasks requiring visual or auditory processing, and reverse-knowledge tests designed to detect AI based on its broader general knowledge (2). Yet the scale of the threat is unknown. A critical question is how much undisclosed AI participation these platforms already carry. Zhang et al. experimentally answered it by evaluating the degree of AI-agent penetration in major survey platforms. They also evaluated the performance of AI detection tools including a standard industry tool [reCAPTCHA v3 (3)], and text typing timing measures. As a control group, they measured the false-positive rate of AI detection among attentive human participants tested in person. This allowed them to robustly estimate the magnitude of the problem in the wild. Results confirmed significant penetration of AI bots in all major online survey platforms, ranging from 6 to 41% across platforms, compared with a false-positive rate of about 2% among verified humans (1), a spread that tracks long-standing differences in data quality across these panels (4). They also show that this contamination can cause biases in answers for survey questions, for example responses flagged as AI rated the ethicality of using AI to complete surveys far more permissively than verified humans did.

The good news is that reputable platforms can still sustain relatively clean ecosystems, and researchers can remove AI agents that managed to penetrate by including efficient detection tests in their surveys. The particularly high rate of problematic behavior (41%) was observed only on the MTurk platform, which has since been discontinued. Commercial agents prompted to take surveys and adversarial agents built by independent experts specifically to evade detection were both caught efficiently. The bad news is that Zhang et al. discovered what it would take for those agents to avoid detection—and it is not much. They commissioned an agent built with full knowledge of the test battery and the ability to iterate against the live survey. This agent could not be detected by any test they attempted. What currently protects us from them is simple economics: Running the agent costs about six times more than participant compensation. This is not very reassuring, given how rapidly the cost of AI agents has fallen in recent years (5).

Zhang et al. suggest that researchers should vary and continually renew their tests, but this is hard because science is built on shared, reproducible methods. The adaptive dynamic here is fast because the detection literature is itself the mutation source: every published battery tells operators exactly which behaviors to suppress. Detection speeds up this process by removing the participants it catches, leaving behind strategies that current tests fail to detect (3, 6). As models are trained on an increasingly large corpora of human behavior, the behavioral gap used for detection will keep shrinking (7).

Although measuring timing of key-strokes are among Zhang et al.’s most effective tests, they remain a software signal that increasingly capable agents will ultimately learn to imitate. A robust solution may need to rely on physical rather than behavioral signals. An agent cannot easily reproduce a human body and the full complexity of adaptive eye movement in front of a sensor. This argues for shifting the burden from AI detectors to video monitoring, eye-tracking systems, and human biometric detectors. A new industry is already emerging, where companies sell human authenticity tools (8). The claim is not that such systems cannot be fooled—deepfaked video and spoofed sensors are active areas—but that fooling them will remain expensive (8). We expect an ongoing interplay between methods for bypassing authentication and methods for detecting and countering these attempts. The resulting “authentication ecosystem” could resemble today’s cybersecurity ecosystem (9), with major providers employing specialized “red teams” to simulate attackers and continuously updating defense tools in response to emerging methods of circumvention.

However, the growing need for verification has social and ethical consequences. Every increment of verification is an increment of surveillance, and the participants most likely to refuse biometric identification are those whom online recruitment was most valuable for reaching: non-WEIRD (Western, Educated, Industrial, Rich and Democratic) populations (10), undocumented workers, political minorities, and anyone with reason to avoid a registry. A verification regime strict enough to guarantee authenticity could reintroduce the sampling bias that online platforms spent two decades dismantling (11). Authenticity is therefore not a test to be passed but an ecosystem to be designed, trading authenticity against privacy and participation. It is a social science problem.

Zhang et al. study one particular type of social-feedback system. In a survey, information is collected post hoc from a separate group of respondents who are explicitly recruited and incentivized to provide feedback. Many real-world feedback systems are more complex. Consider commercial platforms such as Amazon, streaming services such as Spotify, online marketplaces such as eBay, or service marketplaces such as Airbnb. In these systems, the people providing feedback are also consumers and sometimes producers, and their behavior is shaped by different incentives. Feedback is continuously integrated into the system through interactions among consumers, producers, recommendation algorithms, and social networks (12).

AI agents can enter these systems in multiple roles: as bots attempting to shift a market toward a desired state, as agents acting on behalf of individual users, or as producers, evaluators, and recommenders (13). Similar questions arise in collaborative work. In a company’s coding pipeline, a design team, or a creative marketplace, the problem is not simply whether to use AI, but how humans and artificial agents should be combined to achieve collective goals.

Like it or not, society is becoming increasingly human–AI hybrid: Information, decisions, and cultural products now emerge from interactions among populations of humans and artificial agents (14, 15). The central questions are therefore under what conditions human–AI collaboration flourishes, how social feedback can remain sufficiently informative to sustain healthy collective dynamics, which roles should be assigned to humans and AI, and at what mixing ratios. A science of human–AI hybrid societies is now needed (14, 15). The better we understand how human and machine behavior differ, and how each changes when the two interact, the better we can both authenticate agents and judge which mixtures are useful and which are harmful.

**Fig. 1.**
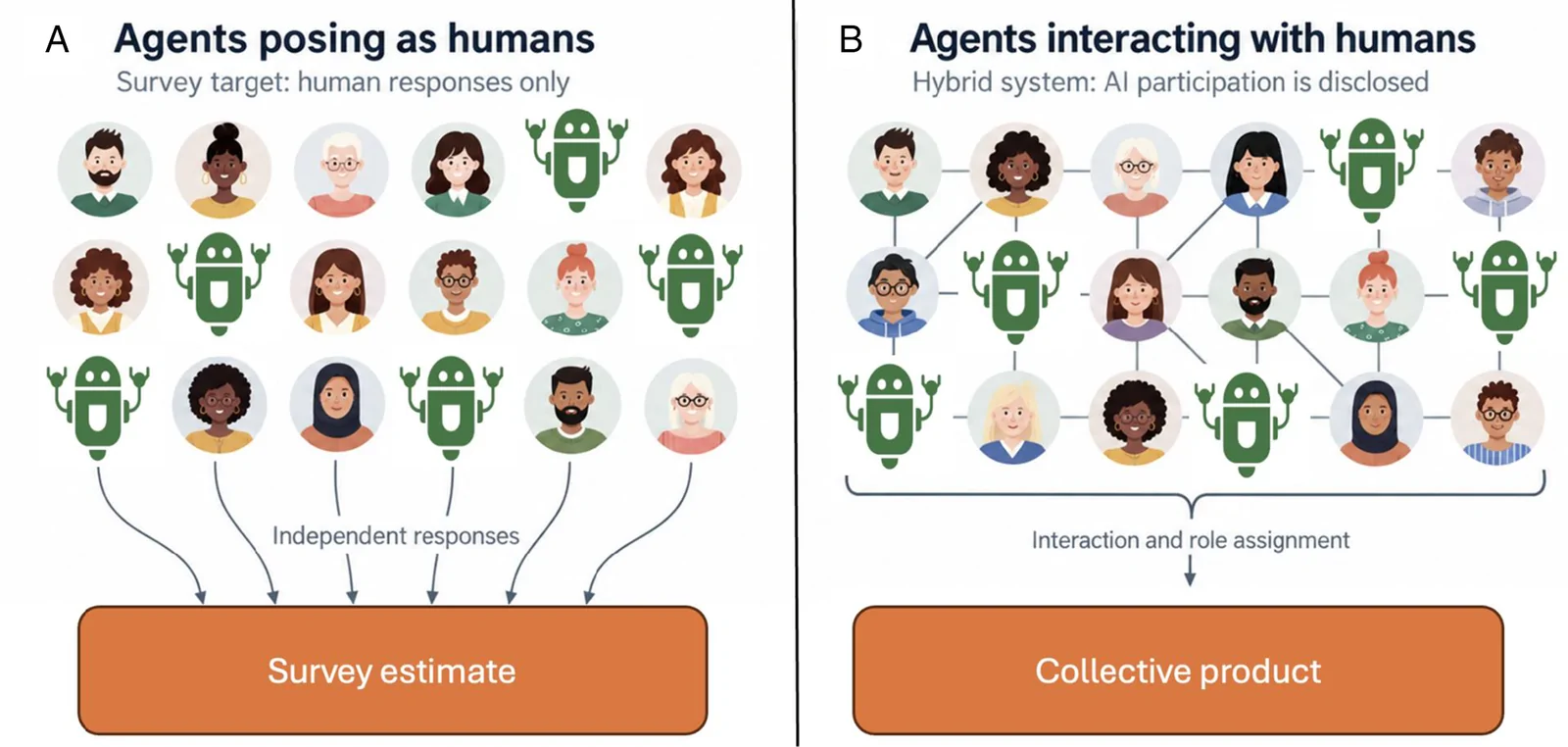
Two regimes of AI participation. (*A*) Undisclosed agents submit responses to a survey intended to measure human judgments, creating risks of bias and homogenization. The design goal is to prevent AI-generated responses from substituting for human judgments. (*B*) In a deliberately hybrid system, humans, and disclosed agents interact to produce a collective output. Outcomes depend on population composition, interaction structure, and role assignment, and may either improve or deteriorate relative to single-population baselines.

Take homogeneity, for example: AI outputs vary far less than human (16) because agents deployed at scale are often nearly identical clones, while no two people are. Where agents substitute for humans, homogeneity can be used for authentication at the population level: as the agent share rises, outputs converge, and convergence can ring alarm bells even if all individual responses look normal. It is an early warning that a system is marching toward detachment (Fig. 1*A*). But in cases where humans and agents interact to create products, the outcomes are no longer additive (14). In some tasks we observe a better-together effect, with a proper balance of quality and diversity exceeding what either population reaches alone (17, 18); in others, mixed populations merely interpolate (19); in still others, they degrade (20). The behavior of both humans and agents may change indirectly by contact (14, 17, 18). The direction of that change is not predictable from the two populations measured separately (Fig. 1*B*). Beyond trivial cases, there is no theory yet that can identify the scope or predict scenarios where adding agents helps, which is why composition has to be measured experimentally.

In PNAS, Zhang et al. (1) document how AI agents posing as humans are already corrupting information systems through which modern societies observe themselves.

AI agents therefore pose an immediate threat to survey science and will likely drive the emergence of an authentication ecosystem that must continually update its defenses against increasingly sophisticated agents. At the same time, AI agents can also improve the quality of social feedback and creative products by contributing synergistically with humans. Future social systems will therefore need not only to defend against AI agents but also to harness them: agents that are transparent and appropriately integrated may improve collective outcomes but they may also undermine them, or become misaligned in unpredictable manners. Understanding when increasing AI participation causes harm and when it produces benefits is a major scientific challenge spanning machine learning, human–computer interaction, sociology, and cognitive science. Identifying these conditions is a necessary step toward mitigating the risks of AI participation while retaining its potential benefits.
